# Alpha/Beta T-Cell Depleted Grafts as an Immunological Booster to Treat Graft Failure after Hematopoietic Stem Cell Transplantation with HLA-Matched Related and Unrelated Donors

**DOI:** 10.1155/2014/578741

**Published:** 2014-10-13

**Authors:** E. Rådestad, H. Wikell, M. Engström, E. Watz, B. Sundberg, S. Thunberg, M. Uzunel, J. Mattsson, M. Uhlin

**Affiliations:** ^1^Department of Oncology and Pathology, Karolinska Institutet, 141 86 Stockholm, Sweden; ^2^Center for Allogeneic Stem Cell Transplantation, Karolinska University Hospital, 141 86 Stockholm, Sweden; ^3^Department of Clinical Immunology and Transfusion Medicine, Karolinska University Hospital, 141 86 Stockholm, Sweden

## Abstract

Allogeneic hematopoietic stem cell transplantation is associated with several complications and risk factors, for example, graft versus host disease (GVHD), viral infections, relapse, and graft rejection. While high levels of CD3+ cells in grafts can contribute to GVHD, they also promote the graft versus leukemia (GVL) effect. Infusions of extra lymphocytes from the original stem cell donor can be used as a treatment after transplantation for relapse or poor immune reconstitution but also they increase the risk for GVHD. In peripheral blood, 95% of T-cells express the *αβ* T-cell receptor and the remaining T-cells express the *γδ* T-cell receptor. As *αβ* T-cells are the primary mediators of GVHD, depleting them from the graft should reduce this risk. In this pilot study, five patients transplanted with HLA-matched related and unrelated donors were treated with *αβ* T-cell depleted stem cell boosts. The majority of *γδ* T-cells in the grafts expressed V*δ*2 and/or V*γ*9. Most patients receiving *αβ*-depleted stem cell boosts increased their levels of white blood cells, platelets, and/or granulocytes 30 days after infusion. No signs of GVHD or other side effects were detected. A larger pool of patients with longer follow-up time is needed to confirm the data in this study.

## 1. Introduction

Today, allogeneic hematopoietic stem cell transplantation (HSCT) is commonly used as treatment for hematological malignancies, immunodeficiencies, and inborn errors of metabolism [1]. Hematopoietic stem cells (HSC) can be collected from numerous sources, notably through aspiration of bone marrow (BM), through apheresis of peripheral blood stem cells (PBSC), or from frozen cord blood units (CBU) [2].

The model donor for HSCT is a human leukocyte antigen- (HLA-) identical sibling. However, only around one third of all patients have access to a suitable sibling donor. With a steady increase in the number of donors in worldwide registries, unrelated donors are increasingly used [3]. Today, long term outcome using unrelated donors is similar to those obtained using HLA-identical siblings [4]. However, while registries now contain more than 19 million unrelated donors, this pool is not sufficiently large to find an HLA-matched donor for all patients. These patients have access to either related HLA-haploidentical donors or umbilical cord blood (UCB) [5].

HSCT is associated with several potentially lethal complications, for example, relapse of the malignant disease, graft rejection, infectious complications, and GVHD, a condition where donor graft cells attack healthy host tissue [6–8]. Higher levels of CD3+ cells in the graft have clearly been associated with increased risk of GVHD, but also superior GVL effect and less infectious complications [9, 10].

To tackle posttransplant complications such as graft failure and relapse, donor lymphocyte infusion (DLI) and stem cell boosts have successfully been used for decades [11–13]. As the use of DLI or unmanipulated graft to boost stem cells is associated with increased risk of GVHD and the exact cellular content is not fully characterized, tailor-made solutions have been sought [12]. One solution has been positive magnetic enrichment of CD34+ HSCs from the graft, initially by Isolex 300i and later by the CliniMACS device [14–17]. As this efficiently removes most CD3+ T-cells, thereby decreasing the risk of GVHD, patients are increasingly susceptible to relapse and infectious complications [18, 19]. In order to more efficiently utilize supportive cells of the graft, CD3/CD19 depletion and later T-cell receptor (TCR) *αβ* depletion have been successfully used prior to HSCT [20, 21].

The majority of T-cells in peripheral blood (95%) express the *αβ* TCR while the rest (5%) express the *γδ* TCR [22]. In contrast to *αβ* T-cells, *γδ* T-cells can be activated in a nonmajor histocompatibility complex- (MHC-) dependent fashion, via natural killer- (NK-) cell receptors and also toll-like receptors (TLR), placing them on the border of innate and adaptive immunity.

After allogeneic HSCT, increased frequency and function of *γδ* T-cells in transplanted patients are associated with a protective role against cytomegalovirus (CMV) reactivation and disease [23]. This is in line with other studies showing expansion and cytotoxic function of CMV-reactive *γδ* T-cells in the peripheral blood of patients receiving renal and lung transplants [24, 25].

In contrast to *αβ* T-cells, *γδ* T-cell activation is not regulated by MHC molecules making them less likely to cause an HLA-dependent GVHD. The *γδ* T-cell subset has also been shown to provide a protective effect against leukemia relapse, making the exploitation of this cell subset an attractive alternative after HSCT [27, 28].

Recently, several groups routinely started to use *αβ* depletion prior to HSCT transplantation [21, 29]. To our knowledge, *αβ* depletion has not been used as a stem cell booster or as DLI after allogeneic HSCT to treat infections or poor immune reconstitution. In this study, five patients were infused after HSCT with *αβ* T-cell depleted grafts. The indication for infusion of *αβ* T-cell depleted graft in all patients was poor immune reconstitution associated with infectious complications.

## 2. Material and Methods

### 2.1. Patient Characteristics

Five patients received a boost of *αβ* T-cell depleted hematopoietic stem cells. The median patient age was 43 years (2–59 years, *n* = 5) with two male and three female patients. Median follow-up time was 249 days (223–331 days). The primary indication for the *αβ* T-cell depleted booster transplantation was secondary graft failure (GF) in all five patients. Secondary GF in this study was defined as initial signs of engraftment with subsequent development of bone marrow hypoplasia requiring frequent transfusions beyond day 60. In addition, the patients should have prolonged neutropenia (<0.5 × 10^9^/L) and thrombocytopenia (<30 × 10^9^/L). All five patients suffered from additional infectious complications prior to the secondary GF which very likely contributed to the poor graft function.

PCR amplification of variable numbers of tandem repeats was used to evaluate donor and recipient chimerism in CD3+, CD19+, and CD33+ cells enriched from peripheral blood using immunomagnetic beads (Dynal, Oslo, Norway) before and after booster infusion [30]. Four patients were full donors before booster infusion in all three lineages. The fifth patient (1567) showed a mixed chimeric pattern in the CD19 lineage (90% recipient cells) before the booster. The CD3 and CD33 lineage were both >90% of donor origin. For all five patients, the original HSCT donor was used for the *αβ* T-cell depleted boost. No regular preconditioning was given to the patients prior to stem cell boost except UPN 1567 who received low dose chemotherapy due to mixed chimerism. Regarding the four PBSC grafts, the donors were treated with granulocyte-colony stimulating factor (G-CSF) (Amgen-Roche, Thousand Oaks, CA) for 4 to 6 days. The dose of G-CSF ranged from 9 to 11.5 *μ*g/kg per day, administered subcutaneously once daily. The BM donor was not pretreated. Detailed transplantation characteristics are summarized in Table 1. This study was approved by the Regional Ethical Committee in Stockholm, Sweden (2012/1940-31/12).

### 2.2. *αβ* T-Cell Depletion with CliniMACS (Miltenyi Biotech)

Cells obtained from apheresis from donors premobilized with G-CSF (*n* = 4) or a buffy coat obtained from bone marrow (*n* = 1) were washed with CliniMACS buffer (Miltenyi Biotech) in a transfer bag by centrifugation at 200 g, 15 minutes with no brake at room temperature (RT). The cells were resuspended up to exactly 95 mL. A volume of 1.4 mL normal human immunoglobulin (Privigen, CSL Behring GmbH) was added and incubated for 5 minutes at RT. One vial of CliniMACS TCR-*αβ* biotin (Miltenyi Biotech) was added to the cells and incubated for 30 minutes on a rocker at RT. The bag with cells was filled with CliniMACS buffer and centrifuged at 300 g for 15 minutes at RT. Next, the cell pellet was resuspended in buffer up to 190 mL and two vials of CliniMACS Anti-Biotin Reagent (Miltenyi Biotech) were added and incubated for 30 minutes at RT. The bag with cells was filled with CliniMACS buffer and centrifuged at 300 g for 15 minutes at RT. The cells were resuspended in 150 mL buffer, counted and separated on the CliniMACS (Miltenyi Biotech). *αβ* T-cells were depleted using the Depletion 3.1 program. Original fraction, precolumn target (cells for infusion), and nontarget (depleted *αβ* T-cells) were analyzed by flow cytometry. Samples from target and nontarget fractions were also collected and stored in liquid nitrogen for subsequent analysis.

### 2.3. Antibodies and Reagents

Cell viability dye 7-AAD and all antibodies except those specifically mentioned were purchased from BD Biosciences (Franklin Lakes, NJ). Antibodies used for analyses on the fractions directly at point of depletion were FITC-labelled anti-*γδ* (11F2, Miltenyi), PE-labelled anti-*αβ* (BW242/412, Miltenyi, Germany), ECD- or FITC-labelled anti-CD45 (J.33, Beckman Coulter), and PC7- or PC5-labelled anti-CD3 (UCHT1, Beckman Coulter). For thawed samples, the B-cell antibody staining panel consisted of FITC-labelled anti-IgM, PE-labelled anti-CD20 (L27), APC- or ECD-labelled anti-CD19 (SJ25C1), Alexa Fluor-700-labelled anti-CD56 (B159), and Pe-Cy7-labelled anti-CD3 (SK7). The NK-cell antibody panel included FITC-labelled anti-CD158b (CH-L), PE-labelled anti-CD16 (3G8), Alexa Flour 700-labelled anti-CD56 (B159), APC-Cy7-labelled anti-CD8 (SK1), Krome Orange/Pacific Orange (KO/PO)-labelled anti-CD4 (13 B82, Beckman Coulter), and V450-labelled anti-CD3 (UCHT1). The T-cell antibody panel included FITC-labelled anti-CD28 (CD28.2), anti-CD69 (FN50), anti-CD94 (HP-3D9), anti-CD56 (NCAM16.2), pan anti-TCR-*γδ* (IMMU510, Beckman Coulter), pan anti-TCR-*αβ* (T10B9.1A-31), anti-CD95 (DX2), anti-PD1 (MIH4), anti-CD107a (H4A3), antiCD38 (HIT2, ImmunoTools), anti-CTLA-4 (A3.4H2.H12, BioSite), anti-CD158b (CH-L), anti-TCRV*δ*1 (TS8.2, ThermoFisher Scientific), anti-TCRV*δ*2 (B6, BioLegend) anti-TCRV*γ*9 (B3, BioLegend), anti-TCRV*α*24 (6B11, BioLegend), anti-CD25 (M-A251), PE-labelled anti-CD27 (L128), APC-labelled anti-CD45RO (UCHL1), APC-Alexa Fluor 700-labelled anti-CD127 (R34.34, Beckman Coulter Inc.), V450-labelled anti-CD3 (UCHT1), KO/PO-labelled anti-CD4 (13 B82, Beckman Coulter), APC-Cy7-labelled anti-CD8 (SK1), PE-Cy7-labelled anti-CCR7 (3D12), and PE-labelled anti-CD39 (TÜ66).

### 2.4. Flow Cytometry, Staining, and Analysis

Flow cytometry on the fresh samples from apheresis, bone marrow, and patients after boost was performed according to standardized protocols at the Karolinska University Laboratory. Briefly, cells were stained with antibodies for 10 minutes and then lysed (IO test lysing solution, Beckman Coulter) for 10 minutes before analysis. The samples were acquired and analyzed on a Beckman Coulter FC500 or Navios using CXP SYSTEM Software.

Cell surface staining of thawed samples was performed as described previously [31]. Briefly, cells were incubated with antibodies for 20 min at 4°C. The cells were diluted and washed in PBS before flow cytometric analysis. Stained cells were acquired on a FACSAria (Becton Dickinson) using FACSDiva software v. 6.1.3. Data was analyzed using FlowJo software (Tree Star, Inc., Ashland, OR). The B-cell subset was defined as CD45+CD3−CD19+ and the NK-cell subset was defined as CD45+CD3−CD56/CD16+.

Blood samples were taken 3–7 months (median 5 months) after boost from four out of five patients for long-term follow-up characterization. The fifth patient succumbed from cerebral hemorrhage. Peripheral mononuclear cells were separated using a density gradient centrifugation with Lymphoprep (Fresenius Kabi, Oslo, Norway). The cells were stored in a nitrogen freezer until analysis. The cells were stained as previously described and were acquired on a FACSCantoII (Becton Dickinson) using FACSDiva software v. 7.0.

### 2.5. Spectratyping of TCR-*γ* and *δ* Chains

cDNA was synthesized from 1.5 *μ*g of RNA using High-Capacity cDNA Reverse Transcription Kit (Life Technologies, Foster City, CA, USA.). Eight multiplex PCR reactions (4 for *γ* and 4 for *δ*) were performed using different labelled primers (Table 3). Primers for spectratyping were adapted from van Dongen et al. [26].

PCR reactions were performed in a total volume of 20 *μ*L including 5 *μ*L cDNA, 200 nM of each primer, and AmpliTaq Gold 360 Master Mix (Life Technologies, Foster City, CA, USA). PCR parameters were as follows: 95°C for 10 min, 35 cycles at 94°C for 30 s, 60°C for 45 s, and 72°C for 60 s. The PCR reaction was ended with an elongation step for 10 min at 72°C.

PCR products were analyzed by capillary electrophoresis on an ABI 3130xl Genetic Analyzer (Applied Biosystems, Foster City, CA, USA). In brief, 4 *μ*L of PCR product was mixed with 10 *μ*L formamide and 0.5 *μ*L ROX-labelled size standard (GS400HD). The DNA was then denatured at 95°C for 3 minutes before analysis by capillary electrophoresis. The results were analyzed with the Peak Scanner program (Applied Biosystems). Gene families were identified based on size and primer label [26].

## 3. Results

### 3.1. Contents of a *αβ*-Depleted Stem Cell Graft

**Figure 1:**
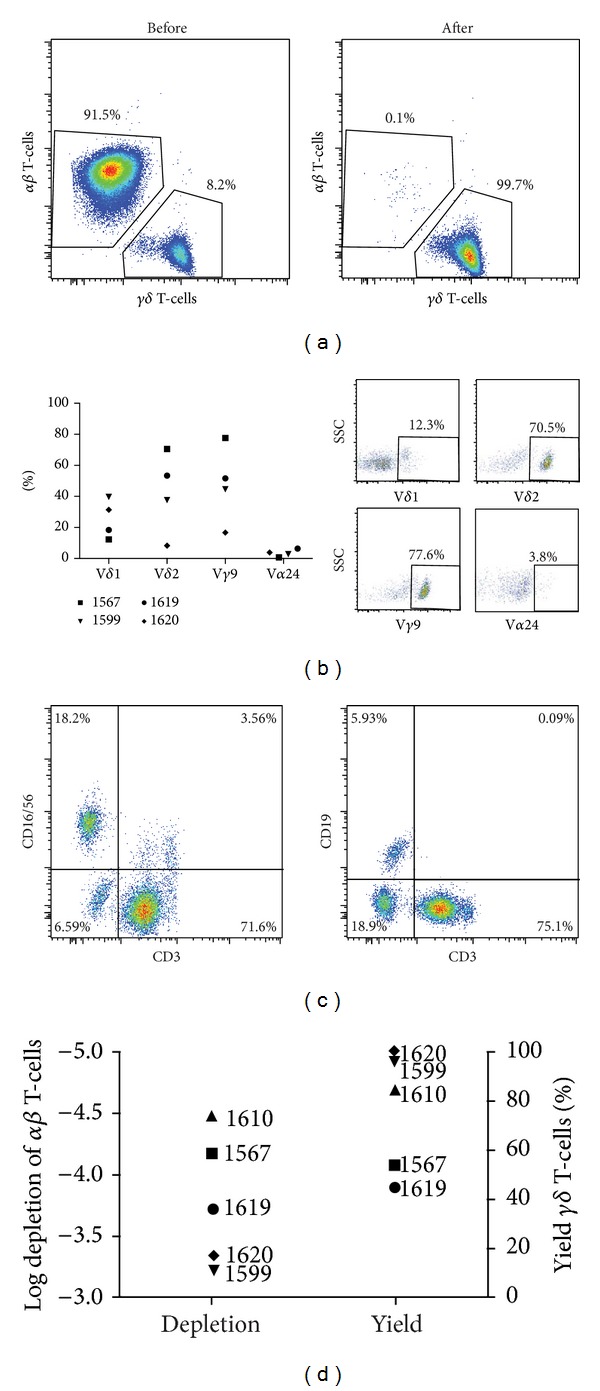
Characteristics of *αβ*-depleted graft. Apheresis products were analyzed by flow cytometry before and after *αβ* T-cell depletion. (a) A representative plot of *γδ* T-cell frequency of the graft before and after *αβ* depletion gated from CD45+CD3+ cells. (b) Analysis by flow cytometry was performed on four out of five *αβ*-depleted graft samples (the sample from the fifth patient had too few cells for analysis). Subgroups (V*δ*1, V*δ*2, V*γ*9, and V*α*24)+ cells of CD3+ T-cells were analyzed and representative plots from the subsets are shown. V*δ*1, V*δ*2, and V*γ*9 subsets are shown from patient 1567 and V*α*24 subset from patient 1620. (c) Representative plots of B- and NK-cells in the graft after *αβ* depletion, gated from CD45+CD19+ or CD45+CD16+/CD56+, respectively. (d) Log depletion of *αβ* T-cells and yield/recovery of *γδ* T-cells after depletion.

To characterize the *αβ*-depleted stem cell grafts, samples were stained for various cellular subsets and analyzed by flow cytometry. As expected, the original apheresis products contained a vast majority of *αβ* T-cells (91.5–98.6%, median 94.6%) when gated on CD3+ cells (Figure 1(a)). The *αβ* depletion was efficient, with only a small population (0.1–1.4%, median 1.02%) of *αβ* T-cells remaining when gated on CD3+ cells (Table 2) (Figure 1(a)). To characterize the enriched *γδ* T-cells in the product, different *γδ* subsets (V*δ*1, V*δ*2, V*γ*9, and V*α*24) were stained for (Figure 1(b)). The grafts contained 12.3–39.7% V*δ*1+ T-cells (median 24.9%). The majority of *γδ* T-cells expressed V*δ*2 (8.3–70.5%, median 45.5%) and/or *Vγ*9 (16.7–77.6%, median 48.1%). A small population of V*α*24+ T-cells remained (0.75–6.4%, median 3.3%) (Figure 1(b)). The depleted grafts contained higher frequencies, compared to normal apheresis or peripheral blood, of B-cells (5.9–17.4%, median 9%) and NK-cells (6–18%, median 7%) (Figure 1(c)). Log depletion of *αβ* T-cells in the products was between −3.24 and −4.46 (median 3.72) and the *γδ* T-cells yield was between 44 and 100% (median 84.4%) (Figure 1(d)).

### 3.2. A Highly Variable *γ*/*δ* TCR Usage between Stem Cell Grafts

**Figure 2:**
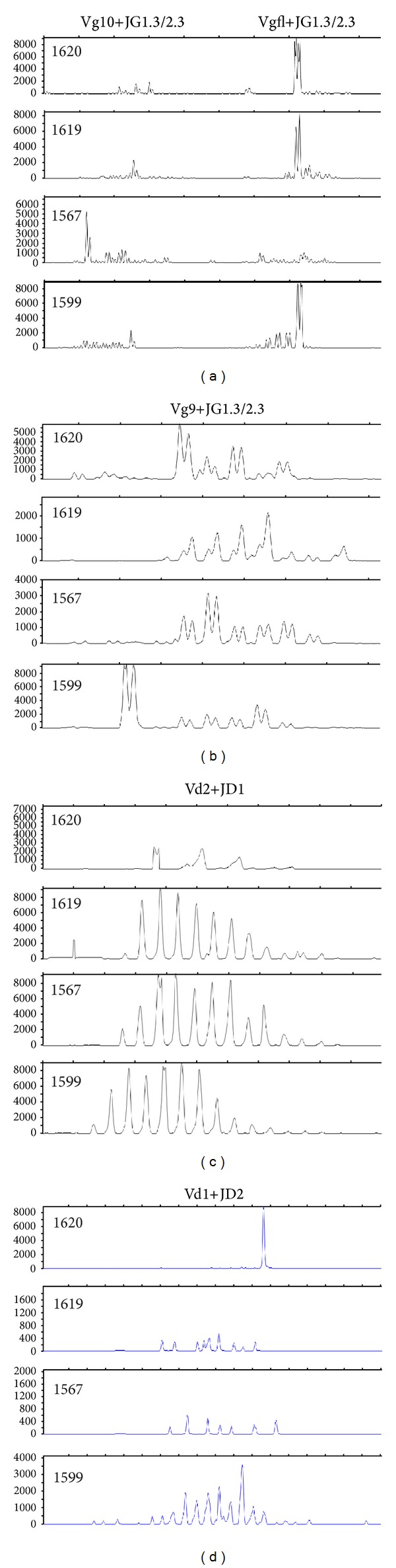
Spectratyping of the *γ*/*δ* TCR. Spectratyping was performed on four out of five frozen graft samples (the sample from the fifth patient had too few cells for analysis). Amplification of (a) Vg10 + JG1.3/2.3 and Vgfl(family) + JG1.3/2.3, (b) Vg9 + JG1.3/2.3, (c) Vd2 + JD1, and (d) Vd1 + JD2.

Variations in greater detail of *γδ* T-cell receptors usage in *αβ*-depleted stem cell grafts were further analyzed by *γδ* TCR spectratyping (Figures 2(a)–2(d)). Only four out of five grafts could be analyzed as DNA was lacking from the graft of patient 1610. In general, the grafts showed a high degree of *γδ* T-cell receptor heterogeneity, both regarding clonality and the use of certain *γ*/*δ* CDR3 combinations. In three out of four grafts, the usage of Vgfl(family) + JG1.3/2.3 was oligoclonal, but of different magnitude; ranging from almost monoclonal (patient 1620) to a more normally distributed oligoclonal pattern (patient 1619). For patient 1567, no sign of normality in the distribution could be observed at all. For Vg10 + JG1.3/2.3, very low levels could be detected in three grafts except in patient 1567, who showed an almost monoclonal pattern (Figure 2(a)). Regarding the usage of Vg9 + JG1.3/2.3, all patients showed an oligoclonal pattern but with different specificities (Figure 2(b)). For Vd2 + JD1 there was a great homogeneity between the grafts with a Gaussian-distributed polyclonal pattern in all grafts (Figure 2(c)). Vd1 + JD2 were completely nonexpressed in two grafts, while being monoclonal in patient 1620 and polyclonal in patient 1599 (Figure 2(d)).

### 3.3. Leukocyte, Platelet, and Granulocyte Reconstitution One Month after Stem Cell Booster

**Figure 3:**
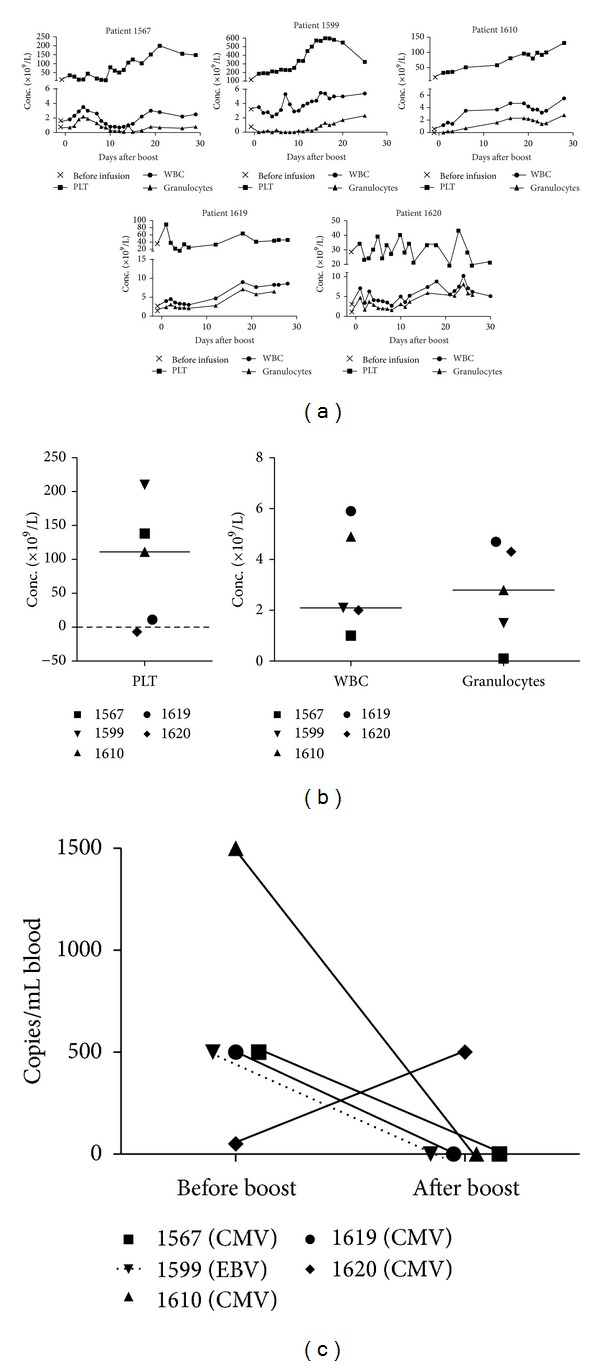
Clinical parameters up to 30 days after infusion of *αβ*-depleted graft. (a) Clinical parameters of platelets (PLT), white blood cells (WBC), and granulocytes of each patient at different time points up to 30 days after infusion. (b) The change in PLT, WBC, and granulocyte concentration (10^9^/L) in the individual patients. Lines indicate the median change in concentration. (c) Cytomegalovirus (CMV) copies per mL blood in four patients andEpstein Barr virus (EBV) copies per mL blood (in patient 1599, dashed line) before and 14–30 days after booster infusion.

Since all patients included in the study suffered from neutropenia and thrombocytopenia the clinical impact of the *αβ*-depleted stem cell booster was assessed by monitoring of platelet (PLT), white blood cell (WBC), and granulocyte counts from the day before infusion and for the first 30 days after infusion (Figure 3). Platelet counts increased in three out of five patients (patients 1610, 1567, and 1599) (median change 111 × 10^9^/L, *P* = 0.1). No difference was observed for patient 1619, while patient 1620 showed a slight decrease in the PLT count (−7 × 10^9^/L). Only a single patient (1599) had a normal PLT count (>150 × 10^9^/L) 30 days after transplant. The WBC count increased gradually in all five patients 30 days after infusion, indicative of a beneficial effect on engraftment (median change 2.1 × 10^9^/L, *P* = 0.06). Four out of five patients (patients 1619, 1610, 1599, and 1620) had a normal WBC count (>3.5 × 10^9^/L). Finally, granulocyte counts increased for all five patients (median change 2.8 × 10^9^/L, *P* = 0.04). Four of the five patients had a normal granulocyte count (>1.6 × 10^9^/L) within 30 days after infusion (patients 1619, 1610, 1599, and 1620). A compilation of the clinical data can be seen in Figure 3(b).

### 3.4. Effect on Infectious Complications

Regarding infectious complications, the stem cell booster may have had a positive effect in all individuals except patient 1620 (Table 1). Of the five patients, 1567 suffered from the least infectious complications. After transplantation, the patient suffered from CMV reactivation with some lung involvement, which later resolved after ganciclovir treatment. Even after antiviral treatment, fluctuating CMV viral copies were observed in peripheral blood until time of the stem cell booster. Two weeks after infusion, viral copies could not be detected (Figure 3(c)). Patient 1599 suffered from an Epstein Barr virus- (EBV-) driven posttransplant lymphoproliferative disease (PTLD) three months prior to stem cell booster. This was successfully treated with four doses of rituximab. One month before infusion, viral copies were detected again in peripheral blood. This together with a severe mucositis due to neutropenia not responding to G-CSF treatment made her candidate for a stem cell booster. As her WBC and granulocyte counts increased (Figure 3(a)), her mucositis resolved and EBV copies became undetectable (Figure 3(c)). Patient 1610 developed cerebral toxoplasmosis after SCT. Due to neutropenia she did not tolerate sulfadiazine prophylaxis. After the diagnosis of cerebral toxoplasmosis it was decided to treat her with pyrimethamine and sulfadiazine together with a stem cell boost in order to increase WBC (Figure 3(a)). One month after booster, she was toxoplasma negative in the blood. Two months later a magnetic resonance image showed progress of the cerebral lesions but without any neurological symptoms. The treatment was changed to high dose clindamycin in combination with pyrimethamine. The patient is still on that treatment and in good clinical condition after the diagnosis of cerebral toxoplasmosis. Patient 1619 suffered from a genital HSV1 infection, which did not respond to acyclovir, cidofovir, or foscarnet due to HSV gene resistance. Prior to the booster she was also tested positive for HSV1 in the blood. The low WBC, platelet, and granulocyte count made her candidate for the stem cell booster. In analogue to patient 1599, her HSV lesions declined as her granulocyte and WBC counts increased (Figure 3(a)). One month after booster, the lesions were gone and she was negative for HSV1 in the blood. In addition, she had detectable CMV copies in the blood, which turned negative after the stem cell booster (Figure 3(c)). Patient 1620 suffered from severe CMV colitis and GVHD grade II in the GI-tract. Only low CMV copies were detectable in blood. The colitis did not respond to ganciclovir, foscarnet, or maribavir. Due to the low WBC (Figure 3) she was considered for a stem cell booster. Except a marginal positive effect on the granulocyte count (Figure 3(a)), no improvement in the colitis or other parameters was observed (Figures 3(a) and 3(c)). No GVHD or other infusion-related side effects were observed in any of the patients after the stem cell booster.

### 3.5. Lymphocytes Frequencies before and after Stem Cell Booster

**Figure 4:**
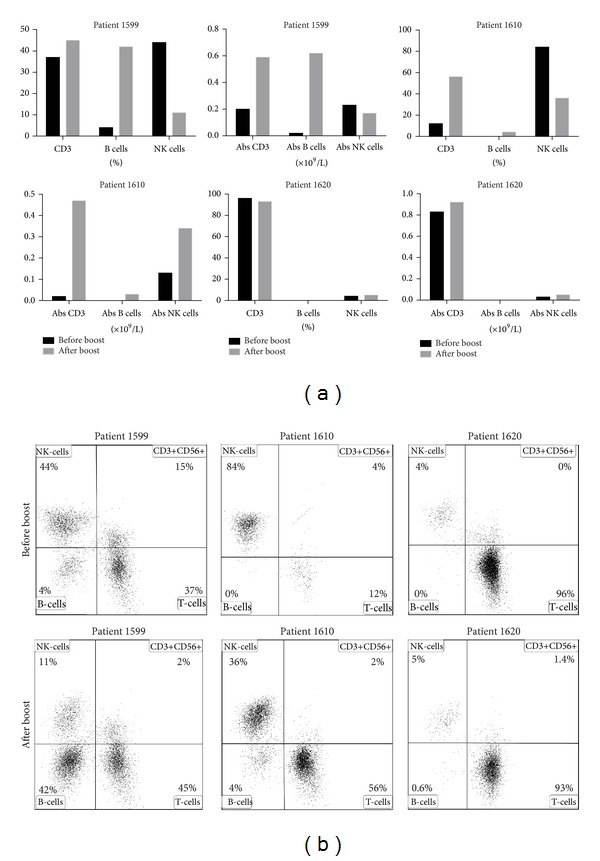
Lymphocyte subpopulations before and after infusion. Blood samples were collected from the patients before and after *αβ*-depleted graft infusion and lymphocyte subpopulations were analyzed by flow cytometry. (a) Both percentages and absolute concentrations are plotted. (b) Representative FACS plots of the three patients before and after infusion. The plots are gated from CD45+ cells.

Routine flow cytometric analysis was performed 2–4 months after infusion on three patients to investigate changes in T-, B-, and NK-cell frequencies and absolute values before and after stem cell boost (Figure 4). Data was missing for two patients due to death (cerebral hemorrhage) and treatment in another hospital. All these three patients were full donors chimeric in CD3+, CD19, and CD33+ lineages both before and after the stem cell booster. The frequency of T- and B-cells increased in two out of three patients with a concomitant decrease in NK-cells (patient 1599 and 1610). A decrease in T-cell frequency and no major differences in B- or NK-cell frequencies were seen in patient 1620 (Figure 4(a)). All three patients had an increase in the absolute numbers of CD3+ T-cells after infusion. In patients 1599 and 1610, the absolute numbers of CD19+ B-cells also increased. In patient 1620, the levels of CD19+ cells were too low to be able to draw any conclusions from. For CD56+CD16+ NK-cells, two patients (1620 and 1610) had an increase in absolute levels while patient 1599 had a decrease.

### 3.6. Characterization of *γδ* Repertoire in PBMCs > 3 Months after Boost

**Figure 5:**
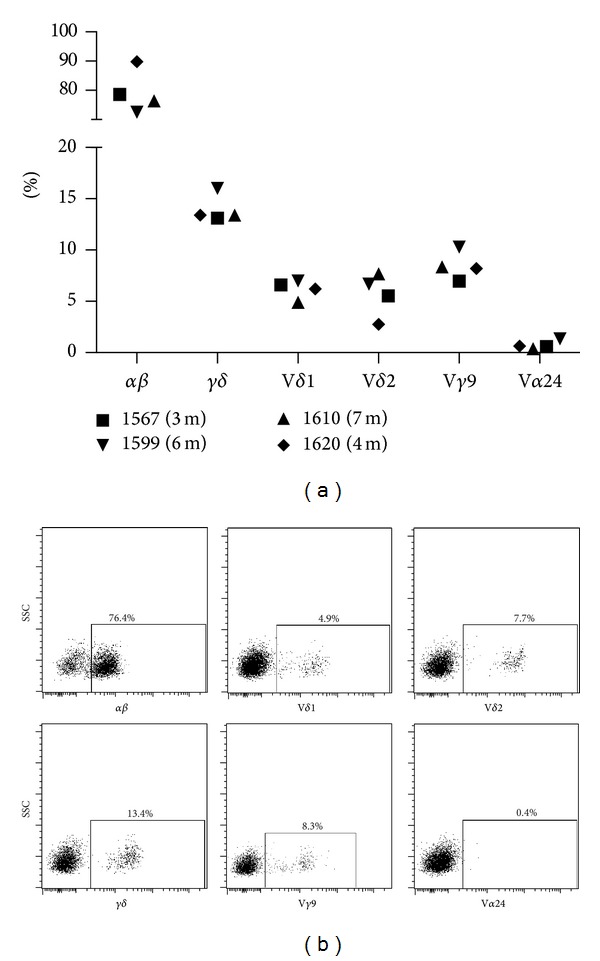
*γδ* subsets in PBMC > 3 months after boost. Blood samples were taken 3–7 months (m) after boost from four patients. (a) Subgroups (*αβ*, *γδ*, V*δ*1, V*δ*2, V*γ*9, and V*α*24)+ cells of CD3+ T-cells from PBMCs were analyzed. (b) Representative plots of the subsets *αβ*, *γδ*, V*δ*1, V*δ*2, V*γ*9, and V*α*24+ cells of CD3+ T-cells from patient 1610, 7 months post boost.

The results of the long-term follow-up blood samples from four out of five patients showed a median of 13.4% *γδ*+CD3+ T-cells (13.1–16.0%) and a median of 77.5% *αβ* T-cells (72.5–89.8%) (Figure 5(a)). The patients had comparable frequencies of the different *γδ* subsets where the expression of V*δ*1 and V*δ*2 was similar (median 6.5%, range 4.9-7% and median 6.1%, range 2.9–7.7%, resp.). The frequency of V*γ*9 ranged from 6.7 to 10.3% (median 8.4%) and the frequency of V*α*24+ was limited in all patients (median 0.6%, range 0.4–1.3%).

## 4. Discussion

HSCT is associated with multiple potential complications after transplantation. The use of DLI is a well-established treatment modality for relapse, rejection, and, in some cases, infectious complications [12, 32, 33]. T-cells are the primary lymphocytes responsible for GVL effects after HSCT, and patients receiving T-cell-depleted grafts are at increased risk of relapse after HSCT [9]. However, *αβ* T-cells are the major cause of GVHD, which can be detrimental to the patient [34]. Thus, depletion of *αβ* T-lymphocytes in DLI products could be beneficial after HSCT.

The infusion of additional donor cells without conditioning (termed “booster”) to treat primary or secondary graft failure has been described by our laboratory and others [13, 35]. Both of these studies reported a significant incidence of acute GVHD after the booster. A booster should be separated from retransplantation where the stem cell infusion is combined with full preconditioning.

Recently, a large-scale clinical method using the CliniMACS TCR *α*/*β* System for selective depletion of *αβ* T-lymphocytes from peripheral blood stem cells has been described [36]. Several ongoing studies with this system investigating facilitated engraftment after MUD and haploidentical transplantation have shown promising results [21, 29]. The CliniMACS TCR *α*/*β* System uses *αβ* TCR-specific, biotin-labelled monoclonal antibodies combined with CliniMACS antibiotin reagent for highly selective depletion. Importantly, selective depletion retains other potential beneficial graft effector cells, such as *γδ* T-cells and stem cells, potentially contributing to engraftment and GVL effects and reducing the risk of infection. To our knowledge, this is the first report using *αβ* T-cell depleted grafts as stem cell booster.

*γδ* T-cells have attracted significant attention in the last decade [37]. T-cells expressing a *γδ* TCR differ fundamentally from conventional *αβ* T-cells. They compose only 1–6% of circulating T-cells but, upon activation in certain infections, can reach up to 50% in extreme cases. *γδ* T-cells play an important role at the verge of innate and adaptive immunity due to the possibility of activation through a MHC-dependent mechanism as well via NK-cells and TLRs. Recently, studies have evaluated the potency of *γδ* T-cells as cancer therapy [38]. After HSCT, increased frequencies and function of *γδ* T-cells in transplanted patients are associated with a protective role against CMV reactivation, disease, and malignant relapse [23, 39].

In theory, *γδ* T-cells may provide an excellent T-cell immunotherapeutic strategy for leukemia after HSCT. This is due to their innate immune effector functions allowing them to respond to malignancies without recognition of alloantigens that could result in unwanted GVHD. In contrast to *αβ* T-cells, *γδ* T-cells directly respond to a variety of MHC-like stress-induced self-antigens overexpressed by a variety of malignant cells.

Here, we report for the first time, the successful use of *αβ* T-cell depleted grafts as a stem cell booster after HSCT. Five patients with infectious complications and inferior immune reconstitution were treated with promising results.

The cell product after *αβ* depletion was characterized in detail by flow cytometry. As expected, a majority of *γδ* T-cells were TCR V*γ*9/V*δ*2, the most commonly V*γ* detected in adult peripheral blood [39]. There was a large spread in the frequency of V*δ*1 cells in the products. This subpopulation is usually more abundant in mucosal tissues than peripheral blood [40]. The two grafts with the highest frequency of the V*δ*1 subset (donors of 1599 and 1620) were both CMV positive in contrast to the other two tested grafts (Table 1 and Figure 1). This observation is in line with previous data showing that CMV activation induces a specific expansion of V*δ*1 and V*δ*3 expressing T-cells [41]. The cumulative frequency of T-cells after depletion expressing either *αβ*, V*δ*1, V*δ*2, or V*α*24 was in all five separations considerably lower than 100% (median 80%) (Figure 1). Earlier studies have shown a significant amount of V*δ*3 expressing T-cells, although being at a much lower frequency than the remaining *γδ* expressing T-cells not positive for the four markers mentioned above would explain [41]. Due to the lack of antibodies available for flow cytometry, it was not possible to determine whether these remaining *γδ* T-cells expressed V*δ*3 or other TCR variants.

The median log depletion of *αβ* T-cells in our study was only 3.7 compared to earlier studies which reported a median of 4.7 [21]. The inferior log depletion seen in our study is probably explained by the fact that four out of five of our products were from MUDs obtained from grafts harvested 1-2 days in advance to processing. This is in contrast to the haploidentical setting where the product is made from fresh grafts.

Spectratyping of *γδ* TCRs was performed in order to further analyze *γδ* T-cell diversity. Interestingly, we could detect a larger heterogeneity between the different graft products than expected (Figure 2). Except for the repertoire profile of Vd2 + JD1, which was polyclonal with a Gaussian distribution for all four grafts (Figure 2(c)), all other tested subsets of *γδ* T-cells were skewed. The majority of tested subsets showed a polyclonal TCR repertoire but with different CDR3 usage between grafts (Figure 2(b)). A clear monoclonal pattern could only be observed in the graft of patient 1620 for Vd1 + JD2 cells (Figure 2(d)). The TCR repertoire of *γδ* T-cells is clearly skewed after HSCT by fungal and viral infections [23, 42]. Peripheral expansions of *γδ* T-cells after renal transplantation and HSCT have also been associated with resolution of CMV infection suggesting they have an antiviral activity [23, 43]. While the repertoire of *γδ* T-cells was so different between the individual grafts, it is tempting to speculate a highly variable effect of different grafts on, for example, infectious complications as seen in these patients. It has earlier been suggested that peripheral expansion of mature T-cells in the graft is the principal pathway of *γδ* T-cell regeneration after HSCT in adults [44]. The correlation of the repertoire of *γδ* T-cells by spectratyping and clinical outcome after HSCT in a larger study would be of great interest.

Few studies have used stem cell boosters as treatment modality after HSCT [13, 35, 45]. In general, patients benefitting from a stem cell booster have a bad prognosis and preferably the treatment should be performed <12 months after HSCT. Using unmanipulated graft as booster, the one-year survival is around 55% or less [13, 35]. Enrichment of CD34+ cells seems to improve outcomes even though the patient data is sparse [16, 35]. Recently a very interesting study on 41 patients treated with enriched CD34+ cells as booster was published with promising results. In the study, a 3-year survival of 63% was reported [17]. The data presented in our report supports further studies into the use of *αβ*-depleted grafts as boosters in order to compare this treatment modality to the existing CD34+ cell enrichment and unmanipulated DLI.

PLT, WBC, and granulocyte counts are commonly used as indicators of successful engraftment. A beneficial effect of the booster was observed in four out five patients on WBC and granulocyte counts 30 days after infusion (Figure 3). A similar positive effect was observed in three out of five patients for platelet counts. Encouragingly, no signs of GVHD were observed in any of the patients and four out five patients were alive six months after infusion. Regarding the indicated infectious complications in the patients, a positive response of the stem cell boost could be observed in all patients except patient 1620 (Table 1). Patient 1619 succumbed from a cerebral hemorrhage. The most obvious response regarding infectious complications could be observed in the three patients with the best combined response in WBC and granulocyte count (Figure 3: patients 1599, 1610, and 1619). As the granulocyte count and WBC increased after the booster in patient 1599, her severe mucositis cleared and EBV copies in peripheral blood vanished. The CMV copies in patient 1610 vanished and her cerebral toxoplasmosis is clinically under control but with continued treatment with clindamycin and pyrimethamine. Due to neutropenia prior to stem cell booster she did not tolerate usual prophylaxis with sulfadiazine. After booster she has tolerated the combined treatment with normal WBC. She turned negative for toxoplasma antigens in peripheral blood after one month. The genital HSV-1 infection in patient 1619 vanished as the WBC and granulocyte count increased (Figure 3(a)) and she also turned negative for HSV-1 antigens in blood as well as undetectable CMV copies (Figure 3(c)). While two of the three patients that turned CMV-antigen negative after the stem cell booster had CMV negative donors (Table 1), the effect on viral clearance more likely is associated with the general hematopoietic reconstitution instead. In patient 1620, no effect on his CMV colitis or CMV antigens in blood could be observed. Neither did his hematopoietic status recover (Figure 3(a) and Figure 3(c)). Until the end of follow-up at 6 months, the patient suffered from several additional bacterial infections. Due to the small patient number, additional studies are required to draw conclusions compared to, for example, boosters with unmanipulated graft or CD34-enriched product.

The frequencies and absolute numbers of T-, B-, and NK-cells 2–4 months after infusion were available for three out of five patients. The use of *αβ*-depleted grafts had a positive effect on the absolute T-cell counts in all three patients (Figure 4(b)). As all these three patients were full donors before and after booster infusion (Table 1) we know that no recipient cells were associated with the lymphocyte development. The positive effect of *γδ* T-cells on total T-cell reconstitution after HSCT has earlier been shown in preliminary studies by the group of Locatelli [29]. We could also observe a positive effect on the total numbers of B-cells in two patients. In the third patient, the numbers were insufficient to analyze (1620). No consistent effect regarding the absolute NK-cell levels could be observed among the patients (Figure 4(b)). Patients 1599 and 1610, which showed the clearest increase in the number of absolute numbers T- and B-cells, also displayed the best effect on their infectious complications (Table 1 and Figure 3(c)). Conversely in patient 1620, no clear increase could be observed in thrombocyte, in WBC (Figure 3), or later in T-, B-, and NK-cells (Figure 4) and there was no improvement in the infectious complications. The analysis of T-, B-, and NK-cells was performed on peripheral whole blood. It is possible that the booster cells after infusion migrated to lymphoid organs to retain homeostasis rather than being circulated in the periphery.

When analyzing the frequencies of different T-cell subsets in four of the individuals (Figure 5) 3–7 months after infusion we could observe that the vast majority of CD3+ cells were *αβ* T-cells in all four individuals, despite the small frequency infused. Later studies might shed light on the kinetics in *αβ* and *γδ* T-cell development close after infusion. Interestingly, regardless of the spread in *γδ* T-cell repertoire in the boost, the expression of the *γδ* T-cell subsets was similar in the four out of five patients whose peripheral blood mononuclear cells were characterized 3–7 months after boost (Figure 5(a)). All seem to have normalized their frequencies of V*δ*1, V*δ*2, V*γ*9, or V*α*24 in blood compared to the infused cell product. Also the *γδ* T-cell population observed in the infused cell product not expressing V*δ*1, V*δ*2, or V*γ*9 seemed to have diminished (Figure 5(a)).

The remaining NK-cells in the booster are most likely to also have a beneficial effect. Alloreactive NK-cells have been shown in the literature to not only have an antileukemic activity but also possibly have a role in preventing GVHD through killing of recipient dendritic cells. They also have an important antiviral function in the immune system [46, 47]. In addition, the effects of the CD34+ stem cells per se should not be neglected. It might be possible that the main part of the positive effects seen on, for example, neutropenia (Figure 3) or lymphocyte reconstitution (Figure 4) is due to only the repopulation of the CD34+ cells in the bone marrow. The individual contribution of *γδ* T-cells, NK-cells, or CD34 stem cells cannot be determined based on this study.

In conclusion, we describe the use of *αβ* T-cell depleted grafts as stem cell booster in five patients suffering from infectious complications due to secondary graft failure after HSCT with encouraging results. No signs of GVHD were observed and four out of five patients were alive six months after infusion. This preliminary data warrants a larger study to compare *αβ* T-cell depleted stem cell boosts with CD34-enriched or unmanipulated grafts.

## Figures and Tables

**Table 1:** Patient characteristics.

Recipient ID	1567	1599	1610	1619	1620
Original HSCT					
Sex of recipient	Male	Female	Female	Female	Male
Age of recipient	2	20	59	43	53
HSCT indication	WASP	Sickle cell anemia	Acute myeloid leukemia	Acute lymphoid leukemia	Lymphoma
Serological status Recipient/donor CMV	+/−	+/+	+/−	−/−	+/+
Serological status Recipient/donor Toxoplasma	−/−	+/−	+/−	−/−	+/−
Serological status Recipient/donor EBV	−/+	+/+	+/+	+/+	+/+
Serological status Recipient/donor HSV1	+/−	−/−	−/+	+/−	−/−
Original graft	BM	BM	BM	BM	PBSC
Preconditioning	RIC	RIC	RIC	MAC	MAC
MUD/related donor	MUD	Sibling	MUD	MUD	MUD
GVHD status	(−)	(−)	(−)	(+)	(+)
Chimerism status prior to booster	MC	DC	DC	DC	DC
Stem cell booster					
Booster indication	Neutropenia thrombocytopenia	Neutropenia thrombocytopenia	Neutropenia thrombocytopenia	Neutropenia thrombocytopenia	Neutropenia thrombocytopenia
Infectious problems	CMV	EBV Mucositis	Toxoplasma	CMV, HSV1	CMV
Source of booster	PBSC	PBSC	BM	PBSC	PBSC
Day of booster	+434	+193	+134	+196	+176
Status 90 days	Alive	Alive	Alive	Alive	Alive
Status 6 months	Alive	Alive	Alive	Deceased	Alive
Response on leucothrombopenia	(+)	(++)	(++)	(+)	(−)
Response on infectious complications	(+)	(++)	(+)	(++)	(−)

WASP: Wiskott Aldrich syndrome, MAC: myeloablative conditioning, RIC: reduced intensity conditioning, MC: mixed chimeric, DC: donor chimeric, MUD: matched unrelated donor, GVHD: graft versus host disease (status prior to booster), CMV: cytomegalovirus, EBV: Epstein Barr virus, HSV: herpes simplex virus, PBSC: peripheral blood stem cells, and BM: bone marrow.

**Table 2:** Graft characteristics.

Recipient ID	1567	1599	1610	1619	1620
Patient weight (Kg)	14,6	73,0	70,5	65,0	70,0
Original product					
*αβ* T-cells (×10^6^/Kg)	930	133	5,5	365	245
*γδ* T-cells (×10^6^/Kg)	83,5	9,3	0,2	4,7	11,1
Volume (mL)	350	427	323	312	355
Target fraction					
TNC dose (×10^8^/Kg)	19,3	24,3	2,6	31,1	15,1
Lymphocytes (%)	19,0	30,0	3,1	4,9	28,3
Lymphocytes (×10^6^/Kg)	251	99,7	1,1	23,5	64,3
CD3+ (%)	19,2	20,0	16,8	9,3	15,3
CD3+ (×10^6^/Kg)	48,2	20,0	6,1	2,2	9,8
*αβ* T-cells (% of CD3+)	0,1	0,4	2,8	1,4	1,0
*αβ* T-cells (×10^6^/Kg)	0,004	0,067	0,005	0,030	0,115
*γδ* T-cells (% of CD3+)	99,7	98,2	88,1	97,3	92,2
*γδ* T-cells (×10^6^/Kg)	45,6	19,9	0,2	2,1	11,1
CD19+ cells (%)	17,2	48,4	49,3	24,5	56,3
CD19+ cells (×10^6^/Kg)	43,2	48,3	0,6	5,8	36,2
CD16+/56+ cells (%)	60,2	29,1	27,6	62,6	27,3
CD16+/56+ cells (×10^6^/Kg)	151	29,0	0,3	14,7	17,6
CD34+ cells (%)	0,7	1,8	1,3	1,2	1,7
CD34+ cells (×10^6^/Kg)	8,7	5,5	0,4	5,2	3,6
Depletion efficiency					
Yield *γδ* (%)	54,6	97,2	84,4	44,2	101
Log depletion	−4,2	−3,2	−4,5	−3,7	−3,3

*αβ*: alpha beta, *γδ*: gamma delta, and TNC: total nucleated cells.

**Table 3:** Multiplex PCR reactions used for spectratyping.

Multiplex	Primer
TCR-*γ*1	V*γ*fI + V*γ*10 + J*γ*1.3/2.3 (NED∗) + J*γ*1.1/2.1 (FAM∗)
TCR-*γ*2	V*γ*11 + J*γ*1.3/2.3 (NED) + J*γ*1.1/2.1 (FAM)
TCR-*γ*3	V*γ*9 + J*γ*1.3/2.3 (NED) + J*γ*1.1/2.1 (FAM)
TCR-*γ*4	V*γ*9 + J*γ*1.2^†^ (FAM)

TCR-*δ*1	V*δ*1 (FAM) + V*δ*2 (NED) + J*δ*1
TCR-*δ*2	V*δ*1 (FAM) + V*δ*2 (NED) + J*δ*2
TCR-*δ*3	V*δ*1 (FAM) + V*δ*2 (NED) + J*δ*3
TCR-*δ*4	V*δ*1 (FAM) + V*δ*2 (NED) + J*δ*4

*Primers were labelled with either NED or 6-FAM.

Primer sequences can be found in [26].

^†^J*γ*1.2 sequence: AAGAAAACTTACCTGTAATGATAAGC.

## Abbreviations

*αβ*: Alpha beta
BM: Bone marrow
CB: Cord blood
CBU: Cord blood units
CD: Cluster of differentiation
CMV: Cytomegalovirus
DLI: Donor lymphocyte infusions
EBV: Epstein Barr virus
GF: Graft failure
GVHD: Graft versus host disease
GVL: Graft versus leukemia
*γδ*: Gamma delta
G-CSF: Granulocyte colony stimulating factor
HLA: Human leukocyte antigen
HSCT: Allogeneic hematopoietic stem cell transplantation
HSC: Hematopoietic stem cells
MHC: Major histocompatibility complex
MUD: Matched unrelated donor
NK: Natural killer
PBSC: Peripheral blood stem cells
UCB: Umbilical cord blood
TCR: T-cell receptor
PLT: Platelets
WBC: White blood cells.
